# Detection and validation of predictors of successful extubation in critically ill children

**DOI:** 10.1371/journal.pone.0189787

**Published:** 2017-12-18

**Authors:** Chiaki Toida, Takashi Muguruma, Masashi Miyamoto

**Affiliations:** Division of Critical Care Medicine, National Medical Center for Children and Mothers, Tokyo, Japan; National Yang-Ming University, TAIWAN

## Abstract

**Introduction:**

Availability of objective criteria for predicting successful extubation could avoid unnecessary prolongation of mechanical ventilation and/or inadvertent premature extubation, but the predictors of successful extubation in children are unclear. This study was performed to detect and validate respiratory function predictors of successful extubation in children admitted to the pediatric critical care unit.

**Methods:**

A retrospective chart review from 2010 to 2012 identified 463 patients, who were divided into a derivation cohort (n = 294) and a validation cohort (n = 169).

**Results:**

The incidence rate of failed extubation was 5% and 9% in the derivation and validation cohorts, respectively. The optimal cut-off values of crying vital capacity (CVC), peak inspiratory flow rate (PIFR), and maximum inspiratory pressure (MIP) were 17 ml/kg, 3.5 ml/sec/cm, and 50 cmH_2_O, respectively. The pass rates of CVC, PIFR, and MIP were 54.2%, 92.7%, and 55.5%, respectively. In the validation cohort, the successful extubation rate was 97.9% for patients who passed all 3 respiratory tests, 88.8% for those who passed at least one test, and 66.7% for those who failed all of the tests. Extubation failed in 5 patients who passed all three respiratory tests and failure was due to postoperative respiratory muscle fatigue or upper airway impairment.

**Conclusions:**

We detected and validated predictors of successful extubation in critically ill children. A combination of CVC, PIFR, and MIP may be used to predict successful extubation for critically ill children. It is necessary to pay attention when extubating patients with postoperative respiratory muscle fatigue or upper airway impairment due to disturbance of consciousness and/or glottal edema even if they pass the respiratory function tests.

## Introduction

Mechanical ventilation is a common treatment for critically ill children admitted to the pediatric critical care unit (PICU). While mechanical ventilation has various benefits, there are also several associated risks. It has been reported that unnecessary prolongation of mechanical ventilation increases the risk of airway trauma, infection, and unplanned extubation [1–2]. It has been reported that the extubation failure rate ranges from 16 to 22% when extubation is performed on the basis of clinical criteria [3–6]. Patients with failed extubation have higher rates of morbidity, mortality, as well as a longer ventilation time and ICU stay [7–9].

Several variables, including respiratory function parameters, may be associated with failed extubation in pediatric patients. However, the criteria for predicting successful extubation in this patient population remain unclear. The availability of objective criteria to predict successful extubation in children could avoid unnecessary prolongation of mechanical ventilation and/or premature extubation.

Therefore, this study was performed to identify and validate predictors of successful extubation in critically ill children admitted to PICU.

## Materials and methods

### Study setting and patient population

This was a single center study, conducted retrospectively from January 2010 to December 2012 in the PICU of the National Center for Children and Mothers (Tokyo, Japan). This PICU has 20 beds and admits an average of 1000 children each year, which is the largest number of critically ill children treated at a single center in Japan.

**Fig 1:**
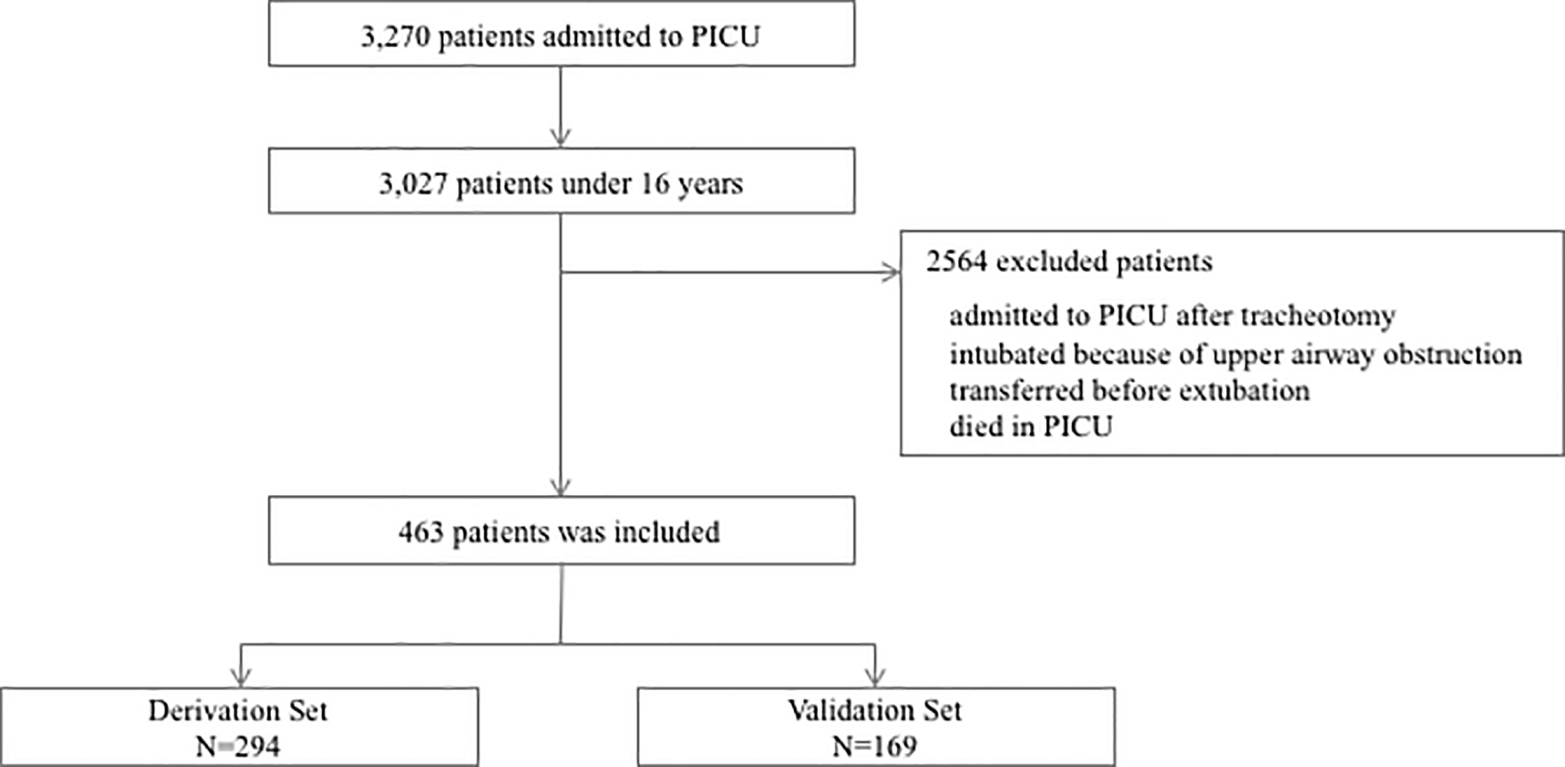
Flow diagram of the study populations. 463 patients were enrolled and were divided into a derivation cohort (n = 294) admitted to PICU from January 2010 to December 2011 and a validation cohort (n = 169) admitted to PICU from January 2012 to December 2012.

The inclusion criteria for this study were (1) an age < 16 years and (2) patients requiring tracheal intubation and mechanical ventilation for > 48 hours. Patients admitted to PICU after tracheotomy, patients intubated because of upper airway obstruction, patients transferred before extubation, and patients who died in PICU were excluded from this study. Accordingly, 463 patients were enrolled and were divided into a derivation cohort (n = 294) admitted to PICU from January 2010 to December 2011 and a validation cohort (n = 169) admitted to PICU from January 2012 to December 2012 (Fig 1).

We used the Newport^TM^ 3260 ventilator. Patients were usually placed on intermittent mandatory ventilation or synchronized intermittent mandatory ventilation in the pressure-control mode. The PICU attending physician weaned patients who fulfilled these criteria: (1) resolution of the primary reason for intubation; (2) no pneumonia, pulmonary atelectasis, or pleural effusion on X-ray films; and (3) the following mechanical ventilation parameters: F_IO2_ ≤ 0.40, peak inspiratory pressure (PIP) ≤ 25 cmH_2_O, pressure support (PS) ≤ 8 cmH_2_O, positive end expiratory pressure (PEEP) ≤ 5 cmH_2_O, and ventilation frequency ≤ 10 /min.

The timing of extubation was determined by the PICU attending physician based on mechanical ventilation parameters, the respiratory rate during spontaneous breathing, and the results of respiratory function evaluation before extubation. The following respiratory function parameters were evaluated with the Aivision respiratory function laboratory (Aivision Corporation, Tokyo, Japan): (1) crying vital capacity (CVC); (2) peak inspiratory flow rate (PIFR); and (3) maximum inspiratory pressure (MIP). We defined failure of extubation as the need for re-intubation and mechanical ventilation within 72 hours after extubation.

We retrospectively validated the predictors of successful extubation using split-sample validation. Therefore, the attending physicians only knew the values of the respiratory function parameters and were blinded to the results of the "all-3-tests" to determine whether extubation should be performed or not.

### Data collection

The following data were collected for each patient: demographic variables [age in months], clinical characteristics [predicted mortality calculated according to the pediatric mortality index2, origin of the patient, primary reason for admission, CVC, PIFR, MIP, and duration of mechanical ventilation before extubation], and outcome information [failed extubation rate, time to re-intubation, duration of mechanical ventilation and PICU stay].

### Statistical analysis

Results are expressed as the median and interquartile range (25^th^–75^th^ percentile) for continuous variables or as percentages for categorical variables. The Mann-Whitney U test was used for analysis of continuous variables and Fisher’s exact test was employed for categorical variables.

The accuracy of using respiratory function parameters to predict successful extubation was evaluated in the derivation and validation cohorts by calculating the area under the receiver operating characteristics curve (AUC) for each parameter. The optimal cut-off value for predicting successful extubation was defined as that achieving the maximum combination of sensitivity and specificity.

Patients were divided into three groups based on the number of respiratory function tests performed successfully: (1) all 3 tests; (2) at least one test; and (3) no tests. Then we calculated the successful extubation rate for each of these groups in the derivation and validation cohorts.

All statistical tests were two-sided and an α value of 0.05 was defined as indicating significance. All statistical analyses were performed using STATA software, version 12.1 (College Station, Texas, USA).

### Ethics statement

The National Medical Center for Children and Mothers Ethics Committee provided consent for the medical record to be used in this study. Data are available from the National Nation Medical Center for Children and Mothers Ethics Committee or researchers who meet the criteria for access to confidential data. The ethics committee waived the requirement for informed consent in individual patients.

There are ethical restrictions on sharing a de-identified data set, because all data including the patient information are owned by the National Medical Center for children and Mothers and the Ethics Committee imposed them.

## Results

### Patient characteristics

Among 3,270 patients admitted to our PICU from 2010 to 2012, 463 patients met the inclusion criteria and their characteristics are shown in Table 1. The primary reasons for admission and total duration of mechanical ventilation differed between the derivation and validation cohorts, but other characteristics did not differ between them. The incidence rate of failed extubation was 5% and 9% in the derivation and validation cohorts, respectively.

**Table 1:** Comparison of clinical characteristics of derivation and validation groups.

Variable	Derivation Set (n = 294)	Validation Set (n = 169)	P value
Age in months, (median, IQR)	11 (4–47)	10 (3–43)	0.088
Predicted mortality*	3.1 (1.5–5.9)	3.3 (1.2–5.7)	0.786
**Patients origin**			
the operating room, n(%)	122 (42)	66 (39)	0.624
the general care floor, n(%)	48 (16)	23 (14)	0.504
the emergency department, n(%)	124 (42)	80 (47)	0.287
**Primary reason for admission**			
Respiratory, n(%)	24 (8)	13 (8)	1.000
Cardiac, n(%)	110 (37)	42 (25)	0.006
Neurologic, n(%)	39 (13)	25 (15)	0.676
Postoperative state of liver transplant, n(%)	26 (9)	29 (17)	0.001
Post cardiac arrest syndrome, n(%)	7 (2)	4 (2)	1.000
Trauma, n(%)	19 (6)	5 (3)	0.063
**Respiration function evaluation before extubation**			
Crying Vital Capacity, ml/kg, (median, IQR)	17.5 (15.4–21.7)	17.0 (15.2–20.2)	0.413
Peak Inspiratory Flow Rate, ml/second/cm, (median, IQR)	4.9 (4.1–5.9)	4.7 (4.0–5.7)	0.686
Maximum Inspiratory Pressure, cmH_2_O, (median, IQR)	51.4 (65.4–42)	52 (66–41)	0.376
Extubation failure, n(%)	14 (5)	16 (9)	0.052
Duration of MV before extubation, days(median, IQR)	5 (4–8)	6 (4–9)	0.234
Total duration of MV, days, (median, IQR)	5 (4–8)	6 (4–10)	0.027
Duration of PICU stay, days, (median, IQR)	9 (6–15)	9 (6–15)	0.376

Abbreviations: IQR, indicates interquartile range (25^th^ -75^th^ percentile); MV, Mechanical ventilation.

*Predicted mortality calculated by the pediatric index of mortality2.

### Detection and accuracy of predictors of successful extubation

The optimal cut-off values of the respiratory function parameters were as follows: 17 ml/kg for CVC, 3.5 ml/sec/cm for PIFR, and 50 cmH_2_O for MIP. Patients with values of CVC, PIFR, and MIP higher than the optimal cut-off value were classified as passing each test. The sensitivity, specificity, positive predictive value (PPV), and negative predictive value (NPV) of CVC, PIFR, and MIP are shown in Table 2. Pass rates for CVC, PIFR, and MIP were 54.2%, 92.7%, and 55.5%, respectively.

**Table 2:** Predictor variables of successful extubation in derivation dataset.

Predictor variables	Optimal cut-off point	Sensitivity (%)	Specificity (%)	PPV (%)	NPV (%)	AUC
CVC, ml/kg	17	57.1	71.4	97.6	7.7	0.60
PIFR, ml/second/cm	3.5	92.1	14.3	95.6	8.3	0.56
MIP, cmH_2_O	50	55.7	50.0	95.7	5.3	0.55

We classified the patients into three groups according to the number of respiratory tests they passed and we compared the successful extubation rate among these groups. In the validation cohort, the successful extubation rate was 97.9% in the group that passed all 3 tests, 88.8% in the group that passed at least one test, and 66.7% in the group that failed all of the tests (Table 3). The PPV and NPV for the “all-3-test” was 97.9% and 12.3%, in the validation dataset.

**Table 3:** Observed incidence rate of successful extubation in the derivation and validation dataset.

group	Derivation dataset			Validation dataset			Total dataset		
	Success, n	Failure, n	Incidence of success, %	Success, n	Failure, n	Incidence of success, %	Success, n	Failure, n	Incidence of success, %
Passed all 3 tests	85	4	95.5	46	1	97.9	131	5	96.3
Passed at least one test	186	9	95.4	103	13	88.8	289	22	92.9
Passed 2 test	133	2	98.5	72	11	86.7	205	13	94.0
Passed 1 test	53	7	88.3	31	2	93.9	84	9	90.3
Passed no tests	9	1	90.0	4	2	66.7	13	3	81.3

**Fig 2:**
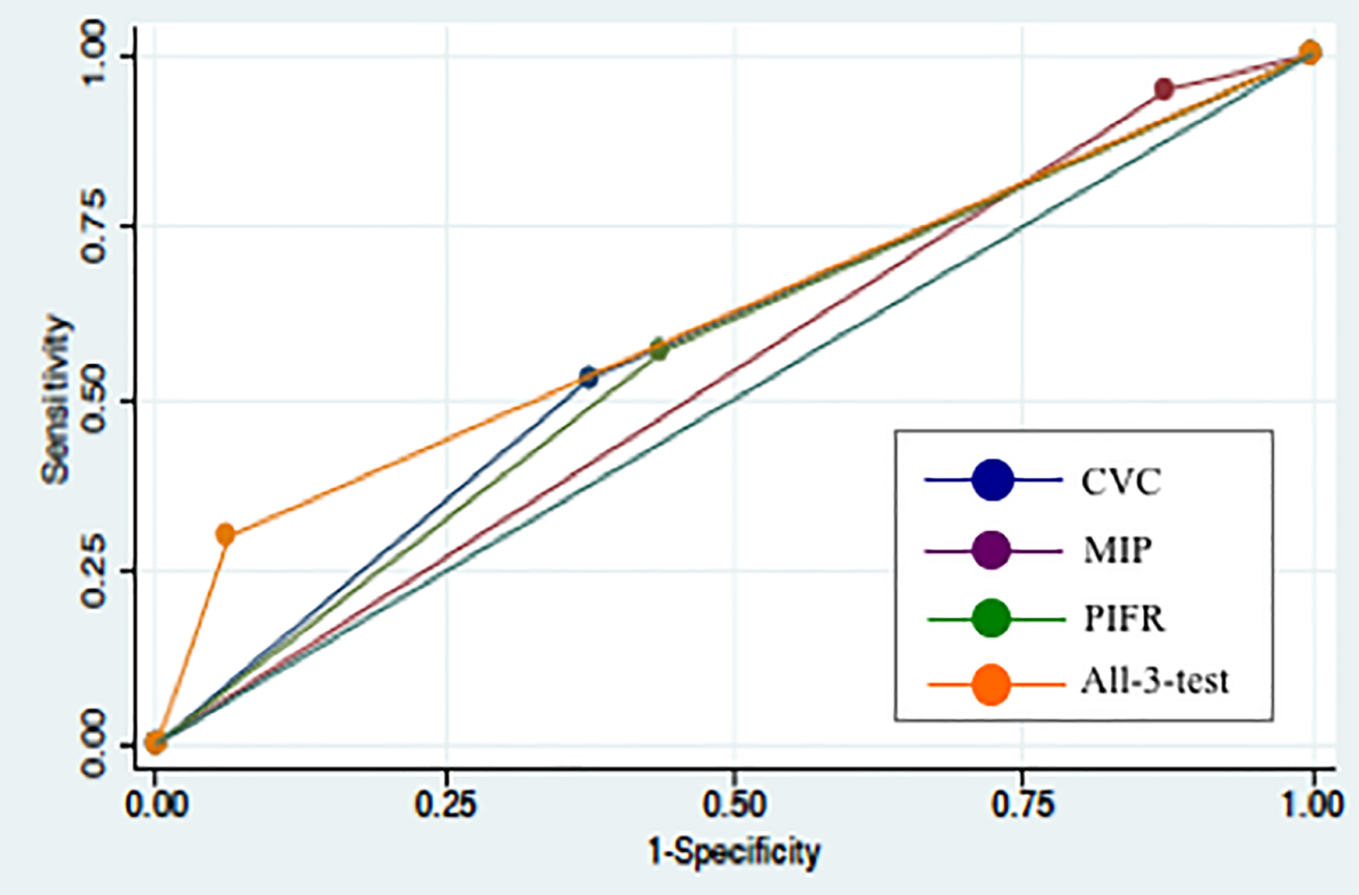
ROC curves for the CVC, PIFR, MIP, and “all-3-test” in validation dataset. The AUC of CVC was 0.58, the AUC of PIFR was 0.54, the AUC of MIP was 0.57, and the AUC of the “all-3-test” was 0.62.

The AUC of each functional parameter and the “all-3-test” in validation dataset was as follows: the AUC of CVC was 0.58, the AUC of PIFR was 0.54, the AUC of MIP was 0.57, and the AUC of the “all-3-test” was 0.62 (Fig 2).

In the group passing all 3 tests from the derivation and validation cohorts, extubation failed in only 5 patients. The clinical characteristics of these 5 patients are listed in Table 4. Four patients were in PICU after surgery and one patient was admitted after cardiac arrest. The reason for re-intubation was respiratory muscle fatigue and deterioration of the respiratory status in 3 patients (Patients 1–3), while 2 patients (Patients 4 and5) had upper airway problems.

**Table 4:** Clinical characteristics of 5 patients with failed extubation in the group passing all 3 tests.

	Age, month	Diagnosis	Predicted mortality*, %	Duration of MV before extubation, days	Time to re-intubation, hour	CVC, ml/kg	PIFR, ml/second/cm	MIP, cmH_2_O
1	4	Postoperative for congenital heart disease	2.4	10	38	20.5	5.9	61
2	6	Postoperative for liver transplantation	17.8	7	70	23.4	6.4	54
3	34	Postoperative for liver transplantation	8.1	13	19	25.8	6.5	61
4	73	Postoperative for pharyngoplasty	0.5	2	9	27.2	4.7	65
5	136	Out-of-hospital cardiac arrest	66.7	7	3	17.6	4.0	75

## Discussion

We investigated the availability of respiratory function parameters as predictors of successful extubation in critically ill children, and we were able to obtain objective criteria for predicting successful extubation based on evaluation of respiratory function. By assessing a combination three variables (CVC, PIFR, and MIP), it was possible to predict successful extubation of critically ill children admitted to PICU.

We determined the optimal cut-off values of CVC, PIFR, and MIP for predicting successful extubation. CVC assesses pulmonary function, while PIFR assesses airway resistance and MIP is an indicator of muscular strength and /or the state of the chest wall and lung. Previous studies in pediatric patients have shown that the CVC value predicting successful extubation was 15 ml/kg in neonates and infants less than 1 year old, while the predictive value of MIP was 45–60 cmH_2_O [10–13]. These values are similar to the results obtained in our study, which were MIP ≥ 17 ml/kg and MIP ≥ 50 cmH_2_O. However, judging from the AUCs for the individual variables, none of these variables demonstrated a high level of accuracy for predicting successful extubation, possibly because our study included children of various ages and with various diseases.

Instead, we demonstrated the possibility of predicting successful extubation in children of various ages with various diseases by evaluating a combination of CVC, PIFR, and MIP. The successful extubation rate was 97.9% in patients who passed all 3 of the respiratory function tests. In other words, the positive predictive value (PPV) of this combination of 3 variables was very high (97.9%). The 5 patients in whom extubation failed even though they passed all of the respiratory function tests had postoperative respiratory muscle fatigue or upper airway impairment due to disturbance of consciousness and/or glottal edema. Therefore, it is necessary to pay attention when extubating patients with these characteristics even if they pass the respiratory function tests.

Another problem is that the successful extubation rate was still a high 88% in patients who did not pass all 3 respiratory function tests. This suggests that we may have to consider another evaluation method in combination with respiratory function tests to overcome the low positive predictive value of respiratory evaluation.

This study had several limitations. First, this study was conducted at a single center in a limited population. It is also essential to research it by using recent data, but in this study we could only evaluate the data of a limited period from 2010 to 2012. Therefore, to generalize the results for larger groups, the study should have involved more participants at different hospitals. Second, a new method is generally validated through 3 steps—derivation, retrospective validation, and prospective validationーbut this study only covered the first 2 steps. Moreover, because the sample size of this study was small, we validated the predictors of successful extubation by using the method of split-sample validation. Therefore, in the future, the prospective validation studies including the design and analysis of prediction models are needed [14]. Third, we did not have the assessment and trial as the objective decision criteria for extubation. Therefore, it is necessary to perform a prospective validation study using a protocol for extubation including respiratory function evaluation. In addition, we did not carry out subgroup analyses stratified by age or the underlying disease.

## Conclusions

We identified and validated three respiratory function tests as predictors of successful extubation in critically ill children. By evaluating a combination of CVC, PIFR, and MIP, successful extubation may be predicted in the PICU setting. Physicians should carefully determine if extubation is appropriate by evaluatinth the results of these tests to prevent premature extubation. Moreover, it is necessary to pay attention when extubating patients with postoperative respiratory muscle fatigue or upper airway impairment due to disturbance of consciousness and/or glottal edema even if they pass the “all-3-tests.”
